# Exploring knowledge, attitudes and practices related to diabetes in Mongolia: a national population-based survey

**DOI:** 10.1186/1471-2458-13-236

**Published:** 2013-03-18

**Authors:** Alessandro R Demaio, Dugee Otgontuya, Maximilian de Courten, Ib C Bygbjerg, Palam Enkhtuya, Janchiv Oyunbileg, Dan W Meyrowitsch

**Affiliations:** 1 Faculty of Health Sciences, University of Copenhagen, Blegdamsvej 3, Copenhagen, DK-2200, Denmark; 2 Copenhagen School of Global Health, University of Copenhagen, Blegdamsvej 3, Copenhagen, DK-2200, Denmark; 3 Public Health Institute, Mongolian Ministry of Health, Olympic Street 2, Ulaanbaatar, Mongolia; 4 Faculty of Health Sciences, University of Copenhagen, Blegdamsvej 3, Copenhagen, DK-2200, Denmark; 5 Public Health Institute, Mongolian Ministry of Health, Olympic Street 2, Ulaanbaatar, Mongolia; 6 Department of Health Services Research, Institute of Public Health, University of Copenhagen, Blegdamsvej 3, Copenhagen, DK-2200, Denmark

**Keywords:** Diabetes, Epidemiology, Gealth policy, Mongolia, Asia, KAP, Knowledge, Population

## Abstract

**Background:**

Non-communicable diseases (NCDs) are now the leading causes of mortality in Mongolia, and diabetes, in particular, is a growing public health threat. Mongolia is a nation undergoing rapid and widespread epidemiological transition and urbanisation: a process that is expected to continue in coming decades and is likely to increase the diabetes burden. To better inform policy and public-health responses to the impact of the growth in NCDs, a national NCD Knowledge, Attitudes and Practices survey was implemented in Mongolia in 2010; a section of which focused on diabetes.

**Methods:**

This survey was a nationally-representative, household-based questionnaire conducted by field-workers. Households were selected using a multi-stage, cluster sampling technique, with one participant (aged 15–64) selected from each of the 3540 households. Questions explored demographic and administrative parameters, as well as knowledge attitudes and practices around NCDs and their risk factors.

**Results:**

This research suggests low levels of diabetes-related health knowledge in Mongolia. Up to fifty percent of Mongolian sub-populations, and one in five of the total population, had never heard the term diabetes prior to surveying. This research also highlights a high level of misunderstanding around the symptomatology and natural progression of diabetes; for example, one-third of Mongolians were unaware that the disease could be prevented through lifestyle changes. Further, this study suggests that a low proportion of Mongolians have received counseling or health education about diabetes, with lowest access to such services for the urban poor and least educated sub-populations.

**Conclusions:**

This research suggests a low prevalence of diabetes-related health-knowledge among Mongolians. In this light, health-education should be part of any national strategy on diabetes.

## Background

Non-communicable diseases, including diabetes, cardiovascular diseases, respiratory diseases and cancers, are now the leading causes of mortality in Low and Middle Income Countries including Mongolia [1]. Independent of the aging population process, this insidious rise in NCDs now poses a threat to economic and social development, as well as population health [1,2].

In 2013, NCDs are no longer only diseases of the rich, the old, and the sedentary. More than 50% of NCD deaths occur in people under the age of 70 years and the majority of these deaths occur in the worlds’ poorest populations [1]. Moreover, it is becoming evident that NCDs, including their risk factors, are reflections of wider social, economic and environmental determinants, rather than simply a result of poor lifestyle ‘choices’ [1,3].

In Mongolia, diabetes mellitus represents a real and growing public health threat. Diabetes prevalence in 2009 was 9% in urban areas and 4% in rural regions. At the same time, pre-diabetes (IFG) was at 12% and 7.3% respectively [4,5]. Mongolia is a nation undergoing rapid and widespread epidemiological transition and urbanisation: a process that is expected to continue in coming decades and is likely to drive the diabetes burden.

Given 80% of global diabetes is believed to be currently preventable, many in the health policy and public health communities call for greater focus, research and investment in known roads to reduce this burden. A crucial first step has been the implementation of national NCD surveillance programs, for example WHO STEPS [6]. These surveys, now implemented in more than 40 nations worldwide, provide accurate, timely and comparable burden data using primarily anthropometric and biochemical parameters. One weakness of STEPS has been the limited focus on the social context of disease, or knowledge, attitudes and practices. This information can be crucial when building health promotion and public health responses [7]. Whether for the appropriate structuring of healthcare systems or health promoting policies, nationally-representative KAPS data may provide governments and health planners more detailed epidemiological information than burden data alone, which could prove crucial for public health decision-making [8,9].

With this in mind, in 2010, the national NCD KAPS survey was implemented in Mongolia designed to complement and triangulate information from the WHO STEPS surveys.

One in a series of publications reporting on the knowledge, attitudes and practices of Mongolians with regards to NCDs, this paper investigates key knowledge, attitudes and practices that may contribute to the national diabetes-related burden. It also aims to shed light on possible policy and prevention ramifications of these findings.

## Methods

The 2010 NCD KAPS survey was a nationally-representative, mixed-methods survey. A full description of the methods has been published [10].

While this survey did not sample the same participants as the 2009 WHO STEPS survey, both methodologies were aligned and both nationally-representative samples were from the same broader population.

Households were selected using a multi-stage, cluster sampling technique, including Probability Proportional to Size sampling (PPS) and simple random sampling from government registries.

The questionnaire was field-worker implemented and administered on one household member from 3450 individual households across Mongolia, in each rural and urban area. Household members, aged 15 – 64 years, were chosen using a predefined algorithm. Questions explored demographic and administrative parameters, as well as knowledge, attitudes and practices around NCDs and their risk factors, and then a more vertical approach surveyed individual disease knowledge, attitudes and practices.

Quality assurance processes included back-translation, pre-testing, piloting and a modified Delphi process of international experts.

Knowledge was probed using a mix of open and closed questions, the first of which asked participants to rate their own level of diabetes knowledge. Participants were asked to volunteer one of four possible ordered responses, from no knowledge, to high knowledge. A series of questions then explored diabetes, the symptomatology and natural progression of the disease, the awareness of its preventable nature and common complications of diabetes. These were single questions posing a statement where the participant was asked to label the statement as true or false.

Prevention and barriers to prevention were then explored. Participants were posed an open-ended question and asked to volunteer prevention methods for diabetes. The most common five responses are reported in this paper. Then participants were asked another open-ended, unprompted question to volunteer main perceived barriers to the consumption of fresh fruits and vegetables. This question was to investigate issues of access as well as knowledge. Similarly, participants were questioned on the perceived barriers to physical activity.

With regards to body weight, in the context of a rapidly increasing prevalence of the overweight and obese in Mongolia, participants were asked to rank the perceived important of maintaining a ‘healthy’ body weight. ‘Healthy’ was left to the participant to define. The participant was asked to rank the importance on a four-level Likert scale, for which a mean and confidence interval were calculated.

Finally, investigating health practices regarding diabetes, participants were asked to report whether a health worker had ever counseled them about diabetes. This was to further assess higher-risk populations when triangulated with prevalence data. For this there was a yes/no response only.

### Ethics approval

This study was conducted according to the principles of the Helsinki declaration. The Mongolian National Ministry of Health’s Medical Ethical Committee approved the study on the 06 October 2010.

### Data analysis

Data input and analysis was performed using the SPSS (IBM SPSS 20.0.0 Statistics) software package. Data was weighted (sample and population weighting coefficients applied) to correct for differences between the sampled population and Mongolian census data. Regression analyses were used to study the presence and strength of associations between populations and their knowledge, attitudes and practices. Major analysis indices included descriptive rates with associated confidence-intervals, as well as univariate and multivariate odds ratios. Covariates included gender, urbanicity, educational attainment and employment status. Age was not routinely included in analyses due to the even age distribution of the sample, and as both education and employment were found to be analogous with age. Significance was assumed at p < 0.05 for all tests (two-sided).

Over-sampling of females occurred due to increased compliance during recruitment (Table 1 versus Additional file 1). Therefore disaggregated data by gender was presented and multivariate analyses were undertaken including gender as a variable.

**Table 1:** Descriptive information on sample population, disaggregated by age, sex, urbanicity, educational level and employment status; Mongolia, 2010

			**Male**	**Female**
		**n (%)**	**n (%)**	**n (%)**
**Total**		3450 (100)	1413 (42.0)	2037 (58.0)
**Age (n=3450)**	15-24	1100 (28)	506 (27.9)	594 (28.0)
	25-34	721 (24.3)	280 (25.8)	441 (23.1)
	35-44	630 (23.0)	234 (22.2)	396 (23.6)
	45-54	507 (19.2)	196 (18.7)	311 (19.7)
	55-64	492 (5.5)	197 (5.4)	295 (5.6)
**Location (n=3450)**	Urban	1737 (50.3)	702 (49.6)	1035 (50.2)
	Rural	1713 (49.7)	711 (50.4)	1002 (49.8)
**Education (n= 3450)**	Primary or less	219 (6.4)	107 (7.5)	112 (5.4)
	Secondary School	2088 (60.5)	919 (65.0)	1169 (57.5)
	Tertiary Schooling	1143 (33.1)	387 (27.4)	756 (37.1)
**Employment (n=3425)**	Student	717 (20.8)	330 (23.7)	387 (18.8)
	Employed	1503 (43.6)	696 (49.9)	807 (39.5)
	Unemployed	508 (14.7)	204 (14.6)	304 (14.8)
	Retired/Home	697 (20.2)	167 (11.8)	530 (25.9)

## Results

This national survey sampled 3450 participants from households across Mongolia. Using a pre-defined algorithm at the final stage of sampling, 42% of participants were male and 58% female. Disaggregated by age, the majority of participants were aged less than 45 years (Table 1). Half of the participants live in the national capital, Ulaanbaatar, in an urban setting. Almost two-thirds of participants achieved high school education only, while a further third attended university. Six percent completed only primary school education, a minority that is evenly spread throughout males and females. Two-fifths of all participants reported to be employed at the time of surveying, with a further one fifth identifying as students. Around 15% were unemployed and 20% reported to be retired or home-makers.

Among Mongolians, one in five reported having never heard the term diabetes prior to being interviewed (Table 2). Significantly more males reported no knowledge than females, as did rural dwellers compared to their urban counterparts. Education was also linked to lower knowledge with least-educated participants five times more likely to lack any knowledge about this disease. This shortfall in awareness is also linked to unemployment. Participants were then asked whether they were aware of asymptomatic early disease (Table 2). One in two Mongolians were aware that diabetes can be asymptomatic in its early stages, and one in three were aware that despite having diabetes it was still possible to lead a ‘normal life’. This health knowledge is linked to urban dwelling and higher educational status, but there are no significant differences between age-groups, sexes, or groups disaggregated by employment. In order to better explore this level of knowledge, questions then probed whether diabetes affects various body organs, including the eyes and vision, the heart and the kidneys. One in three Mongolians were aware of these complications associated with diabetes. Twenty percent more women than men were able to recognise these complications, a significant difference when controlled for other variables. Urban dwellers and higher educated participants were also more likely to be aware of the health risks posed by diabetes. The participants were also questioned on whether diabetes is empirically a preventable disease. Two-thirds of participants were aware that primary prevention of diabetes is possible. This knowledge is higher among urban dwellers as compared to rural counterparts. Education is also a strong predictor for knowledge around the preventability of diabetes, with less than fifty percent of participants in the lowest educated group, versus almost three-quarters in higher educated groups being aware. All these findings are significant, even when other covariates including age are controlled (Table 2).

**Table 2:** Knowledge and attitudes among Mongolians, aged 15–64 years, towards diabetes; Mongolia, 2010

		**Self-Rated knowledge as “Never heard term diabetes before”**		**Aware of possible asymptomatic nature of diabetes**		**Aware that even with diabetes, a normal life can be possible**		**Aware of main health complications of diabetes (heart disease, vision loss)**		**Aware that diabetes is preventable**		**Able to name a diabetes prevention or treatment method**	
		**n (% of Total)**	MOR*	**n (% of Total)**	MOR*	**n (% of Total)**	MOR*	**n (% of Total)**	MOR*	**n (% of Total)**	MOR*	**n (% of Total)**	MOR*
**Total (n = 3450)**		726 (20.5)		1817 (51.3)		1114 (32.4)		1270 (36.9)		2333 (67.7)		2538 (73.6)	
**Gender**	Male	359 (25.4)	1.53	711 (50.4)	1.0	420 (29.8)	1.0	468 (33.2)	1.0	924 (65.5)	1.0	982 (69.6)	1.0
	Female	367 (18.0)	1.0	1106 (54.3)	1.1 (1.0-1.3)	694 (34.1)	1.2 (1.0-1.3)	802 (39.4)	1.2 (1.1-1.4)	1409 (69.2)	1.2 (1.0-1.3)	1556 (76.4)	1.3 (1.1-1.6)
**Urbanicity**	Rural	455 (26.6)	1.7 (1.4-2.0)	848 (49.5)	1.0	521 (30.4)	1.0	567 (33.1)	1.0	1295 (74.6)	1.7 (1.5-2.0)	1192 (69.6)	1.0
	Urban	271 (15.6)	1.0	969 (55.9)	1.2 (1.1-1.4)	593 (34.3)	1.1 (0.9-1.3)	703 (40.6)	1.3 (1.1-1.5)	1038 (60.6)	1.0	1346 (77.6)	1.3 (1.1-1.5)
**Education**	Less than Primary	101 (46.1)	5.2 (3.6-7.3)	89 (40.6)	1.0	49 (22.4)	1.0	56 (25.6)	1.0	103 (47.0)	1.0	100 (45.7)	1.0
	Secondary School	508 (24.3)	2.7 (2.2-3.4)	1056 (50.6)	1.4 (1.0-1.9)	628 (30.1)	1.4 (1.0-2.0)	715 (34.3)	1.5 (1.1-2.1)	1371 (65.7)	1.6 1.4-1.9)	1465 (70.2)	2.4 (2.0-2.9)
	Tertiary	117 (10.2)	1.0	672 (58.8)	1.9 (1.4-2.6)	437 (38.3)	2.0 (1.4-2.8)	499 (43.7)	2.0 (1.4-2.9)	859 (75.2)	2.6 (1.9-3.6)	973 (85.2)	5.6 (4.0-7.8)
**Employment**	Student	128 (17.9)	1.0	369 (51.5)	1.0	228 (31.8)	1.0	202 (28.3)	1.0	522 (72.9)	1.0	526 (73.5)	1.0
	Employed	268 (17.8)	1.3 (1.0-1.7)	805 (53.6)	1.0 (0.8-1.2)	505 (33.7)	1.0 (0.8-1.2)	580 (38.7)	1.5 (1.2-1.8)	1014 (67.6)	1.5 (1.2-1.8)	1152 (76.7)	1.1 (0.9-1.4)
	Unemployed	157 (30.9)	2.2 (1.6-2.9)	258 (50.8)	1.0 (0.8-1.2)	136 (26.8)	0.8 (0.6-0.9)	184 (36.4)	1.5 (1.1-1.9)	319 (62.8)	1.2 (1.2-2.0)	353 (69.5)	1.2 (1.0-1.6)
	Home Maker/Retired	168 (24.1)	1.9 (1.4-2.4)	375 (53.8)	1.0 (0.8-1.3)	241 (34.7)	1.0 (0.8-1.3)	296 (42.5)	1.8 (1.4-2.2)	463 (66.4)	1.6 (1.2-2.0)	494 (70.9)	1.3 (1.1-1.7)

* Multivariate odds ratios, controlled for gender, urbanicity, educational level and employment status.

**Figure 1:**
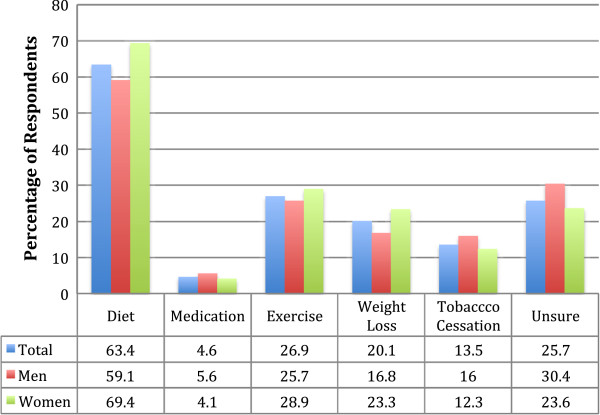
Commonly perceived prevention and treatment methods for diabetes.

In order to confirm knowledge and explore depth of knowledge further, participants were asked to volunteer a prevention or treatment method for diabetes. Examples could include dietary change, weight loss and exercise. More than seven out of ten respondents were able to suggest a prevention or treatment method. This awareness is linked to female sex (MOR 1.3), urban living (MOR 1.3) and higher educational attainment (MOR 5.6), independent of other covariates. The most commonly known prevention methods for diabetes was ‘dietary measures’, an answer given by 63% of respondents, while ‘exercise’ was also common at 27% (Figure 1).

Barriers to prevention methods were then explored. Regarding fruit and vegetable intake, almost two thirds of participants felt that ‘price’ was the main constraint hindering consumption, while one in five felt ‘physical access’ was the main limiter. One in three responded that they did not trust imported foods and that this hindered their fruit and vegetable consumption – a response significantly associated with urban living. For rural populations almost forty percent responded that a lack of access was the main deterring factor to a healthier diet. Perceived barriers to exercise were also studied. Around one third of participants felt that a ‘lack of time’, ‘knowledge’ or ‘interest’ were the main deterrents for Mongolians engaging in more exercise as a means of improving health. There are small differences between men and women; where men were more likely to blame a ‘lack of interest’ and women a ‘lack of time’ or ‘knowledge’.

Finally, participants were asked whether they had ever received counseling from a healthcare professional on the risks or prevention methods for diabetes (Table 3). More than four in five participants have never received any counseling, and counseling attendance is lower in men and in urban areas. Tertiary education is significantly associated with counseling as compared to less educated participants. Unemployed populations are less likely to have received counseling, though this difference was not independently significant.

**Table 3:** Coverage of diabetes-related health counseling services among Mongolians aged 15–64 years; Mongolia 2010

		**Received diabetes counselling from a health worker**	
		**n (% of Total)**	MOR*
**Total (n = 3450)**		528 (15.7)	-
**Gender**	Male	181 (12.8)	1.0
	Female	347 (17.1)	1.3 (1.1-1.6)
**Urbanicity**	Urban	247 (14.2)	1.0
	Rural	281 (16.4)	1.3 (1.1-1.6)
**Education**	Less than Primary	17 (7.8)	1.0
	Secondary School	276 (13.2)	1.7 (1.4-2.1)
	Tertiary	235 (20.6)	3.2 (1.9-5.6)
**Employment**	Student	104 (14.5)	1.0
	Employed	261 (16.8)	1.0 (0.7-1.4)
	Unemployed	52 (10.3)	1.0 (0.8-1.3)
	Home Maker/Retired	107 (16.6)	1.6 (1.2-2.9)

* Multivariate odds ratios, controlled for gender, urbanicity, educational level and employment status.

## Discussion

This research suggests a correlation between populations with higher burdens of diabetes and lower levels of health knowledge. Rural, unemployed, less-educated and male populations are found to hold lower levels of knowledge on diabetes and are the same groups found to experience higher burdens of disease [4].

Findings also suggest that as many as one in five Mongolians has never heard the term ‘diabetes‘. Comparing this finding with other nations in the region, similar studies find analogous results in India where 20% - 25% of citizens are also unaware [8,9,11]. This health-knowledge gap represents a very different public health paradigm for policies and interventions from that in countries such as the USA or Australia where awareness of diabetes is much higher [12]. A precursor for health literacy, this lack of awareness may hinder health promotion strategies currently being implemented in Mongolia, including screening and media campaigns which assume minimum awareness of the disease’s existence [13,14]. This knowledge gap is particularly pronounced among rural participants, both nomadic and small regional town populations. Therefore one could surmise that as the nation continues to experience rapid epidemiological transition and rural to urban migration, a lack of knowledge coupled with changing diets and lifestyle patterns expected, may result in higher burdens of diabetes for future populations [15-17].

Investigating the public’s understanding of diabetes further, this research highlights a high level of misunderstanding around the symptomatology and natural progression of diabetes. On average, just one in two Mongolians are aware than diabetes can be asymptomatic in the early stages of disease. Further, one third of Mongolians are unaware that the disease can be prevented through lifestyle changes and simple medications. While this figure is higher than similar studies in India, this may go some way to explaining the reason 85% of Mongolians have never undertaken screening [8]. These misunderstandings are more prevalent in the male population, of which 90% have never been screened [4].

Finally, this study suggests that a low proportion of Mongolians have received health education regarding diabetes. Those with least access to such services were the urban poor and least educated sub-populations, supporting findings from other research in the Asian region [18]. A recommendation may be the targeted provision of preventative-focused primary care services in areas such as the urban ger districts. Housing the poorest and newly urbanised populations, these districts currently have primary care services though these have a limited role in chronic disease screening and management, which could be expanded [19].

## Conclusion

In conclusion, this research suggests a low level of diabetes-related health-knowledge among Mongolians, with as many as one in two Mongolians having never heard the term ‘diabetes’. This low baseline of health knowledge, crucial to developing population health literacy, may hinder public health interventions particularly as the efficacy of such interventions relies on the pre-existence of basic health-information among target populations [20].

## Competing interests

The authors declare that they have no competing interests.

## Authors’ contributions

AD and DM drafted the manuscript. AD, OD, PA and MdC participated in the design of the study. OD obtained funding for the project. All authors read and commented on manuscript drafts. All authors approved the final draft. All authors read and approved the final manuscript.

## Pre-publication history

The pre-publication history for this paper can be accessed here:

http://www.biomedcentral.com/1471-2458/13/236/prepub

## Supplementary Material

Additional file 1 Mongolian national census data, 2010. Click here for file
