# Supplementary material for: Exploring knowledge, attitudes and practices related to diabetes in Mongolia: a national population-based survey
Source: BMC Public Health. 2013 Mar 18;13:236. doi: 10.1186/1471-2458-13-236 (PMC3606830; doi:10.1186/1471-2458-13-236)
Supplement: Additional file 1 — Mongolian national census data, 2010. [file 1471-2458-13-236-S1.doc]

**Mongolian National Census Data, 2010**

|  | | **2010 National Census Data** |
| --- | --- | --- |
|  | | **n (% of Total)** |
| Total (N) |  | 1,843,285 |
|  | Male | 891,146 (48.3) |
|  | Female | 952,139 (51.6) |
| Age | 15-24 | 579,274 (31.4) |
|  | 25-34 | 475,033 (25.7) |
|  | 35-44 | 387,541 (21.0) |
|  | 45-54 | 275,745 (15.0) |
|  | 55-64 | 125,692 (6.8) |
| Location | Urban | ## |
|  | Rural | ## |
| Education | Primary or less | ## |
|  | Secondary School | ## |
|  | Tertiary Schooling | ## |
| Employment | Student | ## |
|  | Employed | ## |
|  | Unemployed | ## |
|  | Retired/Home | ## |

## Data not available. Source: Mongolian National Institute for Public Health.
